# An Investigation of Outpatient Children's Blood Lead Level in Wuhan China

**DOI:** 10.1371/journal.pone.0095284

**Published:** 2014-04-16

**Authors:** Ying Li, Siqi Wu, Yun Xiang, Xiaohui Liang

**Affiliations:** 1 School of Public Health, Wuhan University, Wuhan, China; 2 Wuhan Children's Hospital, Wuhan, China; Old Dominion University, United States of America

## Abstract

**Objective:**

Blood lead levels (BLLs) and possible influencing factors in children in Wuhan China were investigated in order to understand current lead pollution exposure and provide a scientific basis for prevention and policy making.

**Materials and Methods:**

BLL data were collected from 15,536 out-patients in Wuhan Children Hospital in 2012 full year. All of them were under 18 years of age (Mean ± SD: 4.32±3.2, 64.4% boys). The BLLs were measured by an atomic absorption spectrometry (BH2100).

**Results:**

The geometric mean of BLLs for all the subjects was 44.75 µg/L (95%CI: 44.46 µg/L – 45.05 µg/L), much lower than that reported in previous studies. The prevalence of the elevated BLLs (≥ 100 µg/L) in the children tested was 2% in 2012 and the prevalence of BLLs (≥ 50 µg/L) was 44%. Age and sex could be possible influencing factors for BLLs in the children (p<0.001). In addition, the BLLs in different seasons were different (p<0.001).

**Conclusions:**

These results demonstrate that BLLs have significantly decreased in children in Wuhan during recent years. However, we should continuously pay attention to lead pollution and emphasize that prevention is much more important than treatment for controlling children's BLLs.

## Introduction

Lead is a neurotoxic heavy metal with no known physiologic role in the human body. The ideal human body's blood lead concentration is zero. The potential dangers of lead poisoning have been studied for a long time. As a kind of multi-system and multi-affinity poison, lead can affect children's growth and development, and damage psychological, behavioral and intellectual development. The harm of lead poisoning in children is mainly in functional change of the central nervous system, and there is no safe threshold of lead on children's brain development [1]–[4].

Leaded gasoline and industrial pollution are major sources of lead pollution in the air and soil. In addition to the lead in the air and dust being inhaled through the respiratory tract, daily child behaviors (e.g., chewing or mouthing toys, sucking on fingers, playing on the floor, incomplete hand washing) and dietary habits (e.g., eating canned foods and beverages) may also contribute to lead poisoning as these behaviors may introduce lead directly into the digestive tract. Sometimes, people do not realize the hazard of low dose lead exposure, so lacking public health education is another reason for elevated blood lead levels (BLLs).

Previous studies suggest that BLLs less than 100 µg/L can be associated with neurologic injuries [5], [6]. The Centers for Disease Control and Prevention (CDC) in US defined elevated blood lead level as 100 µg/L or above in children in their 1991 report ‘Preventing Lead Poisoning in Young Children' [7]. Recently,the CDC Advisory Committee on Childhood Lead Poisoning Prevention (ACCLPP) in US recommended elimination of the usage of the term “blood lead level of concern”, because many studies demonstrated that there is no safe BLLs in children [8]. Currently, 50 µg/L in blood is recommended as a level for treatment.

In China,there have been many studies on lead poisoning in children [9]–[12]. In 2006, Wang et al. analyzed the data on BLLs from 32 articles published from 1994 to 2004 in China, and found that the mean BLL of the children was 92.9 µg/L, and 33.8% (9.6–80.5%) of the subjects had BLLs higher than 100 µg/L [13]. In addition, they reported that the mean BLL of the boys (96.4 µg/L) was significantly higher than that of the girls (89.4 µg/L). They also demonstrated that the BLLs increased with age, and those children living in industrial and urban areas had significantly higher BLLs. In 2009, the same group collected data from other 35 articles about children's BLLs published from Jan 2004 to Aug 2007, and found that the mean BLL of the children was 80.7 µg/L (45.5–165.3 µg/L), and 23.9% (3.2%–80.7%) of the children had BLLs higher than 100 µg/L. It appeared that both BLLs and prevalence rate of lead poisoning were lower than those reported in previous studies [14]. For Wuhan, a central city of China, Zhang et al. investigated the BLLs for 5,031 out-patients in 2005, and found that the mean BLL of the children was 72.5 µg/L, and 17.43% of the subjects had BLLs higher than 100 µg/L [15].

Wuhan is a big city located in the center of China, and has developed very quickly during recent years. However, the current status of BLLs and childhood lead poisoning rate in Wuhan is unknown. In this study, we evaluated the current situation of BLLs for children and possible influencing factors in Wuhan in order to support public health action, including public health education to improve individual understating of the risks and promotion of preventive action, as well as changes to the policies to address lead exposure in the community level.

## Materials and Methods

### Ethics statement

For all the children subjects from Wuhan Children Hospital in this study, their parents or guardians had been informed of the participation in this project during clinic diagnoses and their verbal consents were obtained. All the samples were sent to the clinic laboratory to detect the BLLs, and the BLLs for the outpatients were documented anonymously, so the written consents were not sought. The institutional review board of Wuhan Children Hospital had approved the consent procedure and the project.

### Study population

We collected BLLs data from all the pediatric out-patients in Wuhan Children Hospital in 2012 full year, and the average age of the children in this study is 4.32±3.2 (mean ± SD) years.

### Blood lead level determination

For all the patients, 40 µl of the finger blood was moved into the diluents for blood lead determination by unleaded capillary, mixed immediately and kept in 4°C degrees refrigerator for analysis. The blood lead level was analyzed using an atomic absorption spectrometry (BH2100). The spectrometer, reagents and standard solution were all provided by Beijing Bo Hui Optoelectronic Technology in China. Blanks and internal quality controls were used for quality controlling. The sample measurement was carried out in accordance with the instructions for the instrument.

### Lead poisoning criteria

Based on CDC classifications for blood lead concentration screening for children in US in 1991 and the latest blood lead level criteria, we divided the children's blood lead level into six classes: class I: normal level (<50 µg/L); class II: reported level (50–99 µg/L); class III: mild lead poisoning (100–199 µg/L); class IV, moderate lead poisoning (200–449 µg/L); class V: severe lead poisoning (450–699 µg/L), class VI: extremely severe lead poisoning (higher than 700 µg/L). In this study, we used the new criteria 50 µg/L as the cut-off point. In order to compare with previous studies, we took 100 µg/L as the threshold for mild lead poisoning in statistic analysis.

### Statistical analysis

SPSS (version 19) was used for statistical analysis. Since BLLs were not normally distributed, the BLLs were logarithmically transformed in order to approximate a normal distribution. According to central limit theorem, the logarithmic values of BLLs can be taken as normal distribution. The Student's t-test was used to compare the continuous variables between sexes. The mean BLLs were compared among the age-groups and season-groups using one-way analysis of variance (ANOVA). A threshold of 50 µg/L or 100 µg/L for BLLs was used to categorize blood lead levels into normal and abnormal groups. Chi-square analysis was taken to compare categorical variables. Furthermore, logistic regression was used to estimate odds ratios (OR), 95% confidence intervals (CI) and p-value adjusted for potential confounding factors including age, sex and season. Spearmen correlation analysis was examined for the association between BLLs and age. All comparisons were two-tailed, and a p-value < 0.05 was considered as statistically significant.

## Results

Table 1 shows the demographic characteristics of the subjects in this study. The total number of the subjects was 15,536, and the average age of the children was 4.32±3.2 years, ranging from one month to 17 years. The numbers of boys and girls were 10,006 and 5,530 respectively, and there were more boys (64.4%) than girls (35.6%) in the study (Table 1). The possible explanation for the difference was that boys usually are more active than girls, and may be more likely to be suspected as lead poisoning and asked to take BLL test by clinical physicians. In addition, 83% of the subjects were preschool children, and more than one third of the outpatients came to the hospital to check the BLLs in summer, as shown in Table 1.

**Table 1:** Demographic characteristics and comparison of BLLs.

	Number	Frequency (%)	BLLs (µg/L)				P-value
			GM (95%CI)	Median	Min	Max	
**Overall**	15536	100%	44.75 (44.46 – 45.05)	47	3	538	
**Sex**							**p<0.001**
**Boys**	10006	64.4%	46.10 (45.73 – 46.49)	48	4	519	
**Girls**	5530	35.6%	42.41 (41.95 – 42.88)	45	3	538	
**Age (years)**							**p<0.001**
**0–3^a^**	8493	54.7%	41.85 (41.46 – 42.25)	44	3	538	a *vs* b p<0.001
**4–7^b^**	4403	28.3%	48.32 (47.77 – 48.88)	51	5	398	a *vs* c p<0.001
**8–11^c^**	2164	13.9%	49.29 (48.54 – 50.06)	51	6	324	a *vs* d p<0.001
**12–18^d^**	476	3.1%	47.11 (45.49 – 48.80)	49	8	155	c *vs* d p<0.05
**Seasons**							**p<0.001**
**Spring^e^**	3905	25.1%	42.54 (41.95 – 43.15)	45	5	279	f *vs* e p<0.001
**Summer^f^**	5360	34.5%	48.65 (48.16 – 49.14)	50	5	538	f *vs* g p<0.001
**Autumn^g^**	3970	25.6%	43.17 (42.64 – 43.71)	45	4	519	f *vs* h p<0.001
**Winter^h^**	2301	14.8%	42.75 (41.94 – 43.57)	45	3	311	

There were 15,536 out-patients in this study. Based on sex, age and date of measuring BLLs, they were divided into different sub-groups. Student's t-test and ANOVA were used to compare BLLs among different subgroups. There was significant difference in all the sub-groups in statistics (p<0.001).

**Figure 1:**
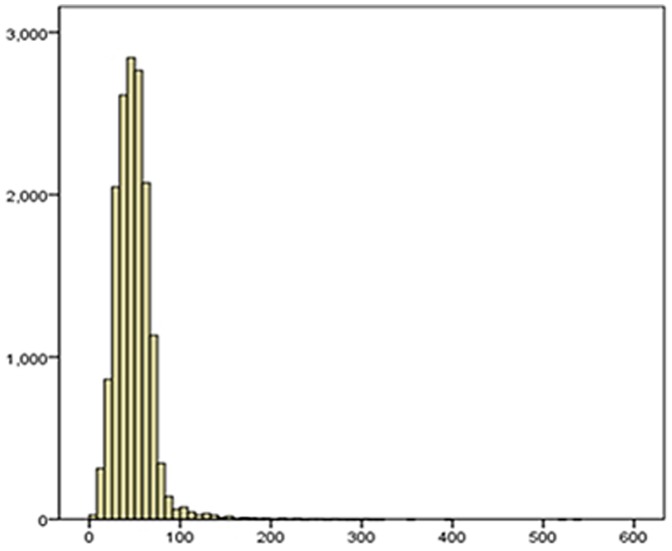
Distribution of blood lead levels among the out-patients in Wuhan. The horizontal axis represents the BLL values, and the vertical axis represents its frequency. Most of Children had BLLs below 100 µg/L, and only 310 children had BLLs higher than 100 µg/L.

Figure 1 is a histogram showing the distribution of the BLLs among the out-patients. The BLL data did not follow a normal distribution, and the P-value for K-S test was less than 0.05 (P<0.001). Figure 1 indicates that most of the children's BLLs were around 40–60 µg/L and below 100 µg/L. In fact, there were only 310 children whose BLLs are higher than 100 µg/L (Figure 1).

The comparison of BLLs in different subgroups including sex, age and BLL measuring seasons is also shown in Table 1. The geometric mean (GM)of BLLs of all the subjects was 44.75 µg/L (95%CI: 44.46 µg/L – 45.05 µg/L), ranging from 3 µg/L to 538 µg/L, and the median was 47 µg/L. Statistical analyses shows that the boys (46.10 µg/L; 95%CI: 45.73 µg/L – 46.49 µg/L) had significantly higher BLLs than the girls (42.41 µg/L; 95%CI: 41.95 µg/L –42.88 µg/L) (p<0.001).

According to the age stages in pediatrics, all the subjects were divided into four age groups: 1–3 years old (toddlers); 4–7 years old (preschool age children); 8–11 yeas old (school age children) and 12–18 years old (teenagers). The results show that the geometric means of BLLs in these four groups were: 41.85 µg/L, 48.32 µg/L, 49.29 µg/L and 47.11 µg/L respectively. ANOVA was used to analyze the difference among the different age subgroups. The results show that there was significant difference between toddlers group and the other three groups (p<0.001), and the BLLs for the toddlers were obviously lower (Table 1). On the other hand, as the age increases, an increasing trend in BLLs (<12 years old) was observed. The Spearman correlation between the age (<12 years old) and the blood lead levels was performed, and a positive correlation was found with high significance (r = 0.23, p<0.001). In addition, the preschool children were further grouped year by year in order to understand the BLL distribution with ages, and a similar result was found that there was correlation between BLLs and ages (<7 years old). For the toddler group, especially the infant group younger than 1-year-old, their BLLs are relatively low (p<0.001) (Table 1).

In order to consider the impact of the climate factor, all the subjects were divided into four groups based on their BLL measuring date in hospital: March to May (Spring), June to August (Summer), September to November (Autumn), December to February (Winter). The geometric means of BLLs from spring group to winter group were 42.54 µg/L, 48.65 µg/L, 43.17 µg/L and 42.75 µg/L respectively. The data shows that a significant difference in BLLs existed among different seasons (p<0.001), and an obvious statistical difference came from the data of summer group. The multiple comparison result in Post Hoc Test demonstrates that the BLLs of summer were significant higher than those of other seasons (Table 1).

According to the lead poisoning criteria, we divided all the out-patients into five groups: group I (<50 µg/L), group II (50–99 µg/L), group III (100–199 µg/L) group IV (200–449 µg/L) and group V (450–699 µg/L), and the numbers of the subjects in these groups are 8,706 (56%), 6,520 (42%), 270 (1.7%), 38 (0.2%) and 2(0%) respectively. According to the previous criteria (100 µg/L) defined by US CDC in 1991, only 2% of the children can be considered as lead poisoning. Recently, CDC announced a new reference level (50 µg/L) for children's blood lead levels, so both BLL categories (> = 50 µg/L) and (> = 100 µg/L) were taken as high lead checking points in statistics. Among the 15,536 children, 6,830 of them had BLLs higher than 50 µg/L (44.0%), and 310 of them had BLLs higher than 100 µg/L (2.0%).

Table 2 shows that if taking 50 µg/L as the criteria, 47% of the boy group and 38.5% of the girl group had higher BLLs than the criteria. Chi-square analysis indicates that the boy group had higher rate for high BLLs than the girl group (p<0.001). It also suggests that significant difference of BLLs exists in different age groups and in different season groups (p<0.001). As shown in Table 2, 52.4% of the children in 4–12 year group had higher BLLs than 50 µg/L, but in the group (<3 year), only 37.2% of the children had BLLs higher than 50 µg/L. The rate with BLLs higher than 50 µg/L was significantly higher in preschool and school children. As to the seasons, 52.8% of the children in summer had high BLLs as compared with other seasons (p<0.001). Furthermore, we used logistic regression to estimate odds ratios (OR), 95% confidence intervals (CI) and p-value adjusted by sex, age and season, and the analyse indicated that BLLs were associated with sex (OR 0.75, 95% CI 0.70–0.80), age (4–7 year group: OR 1.81, 95% CI 1.68–1.95; 8–11 year group: OR 1.72, 95% CI 1.56–1.90; 12–18 year group: OR 1.47, 95% CI 1.21–1.77) and season (summer: OR 1.71, 95% CI 1.58–1.87), and p value was the same as that in Chi-square analysis (Table 2). For the group with BLLs higher than 100 µg/L, a separate logistic regression analysis indicated that girl group had lower possibility for having high blood lead level (OR 0.54, 95% CI 0.41–0.70) and the p-value was less than 0.001 (Table 2). Actually, among the 15,536 children, only 310 had BLLs higher than 100 µg/dL (2.0%). Compared with previous studies (14), the rate with BLLs equal or higher than 100 µg/L in Wuhan obviously decreased.

**Table 2:** High BLL rate at two cut-off points (50 µg/L and 100 µg/L) based on different factors.

Groups	>50 µg/L		OR	95%CI	p value	>100 µg/L		OR	95%CI	p value
	n	Rate(%)				n	Rate (%)			
**Sex**										
Boys	4699	47.0%	1			239	2.4%	1		
Girls	2131	38.5%	0.75	0.70–0.80	**p<0.001**	71	1.3%	0. 54	0.41–0.70	**p<0.001**
**Age (years)**										
0–3	3157	37.2%	1			155	1.8%	1		
4–7	2305	52.4%	1.81	1.68–1.95	**p<0.001**	107	2.4%	1.28	0.99–1.64	p = 0.06
8–11	1135	52.4%	1.72	1.56–1.90	**p<0.001**	41	1.9%	0.95	0.67–1.34	p = 0.76
12–18	233	48.9%	1.47	1.21–1.77	**p <0.001**	7	1.5%	0.72	0.33–1.54	p = 0.40
**Seasons**										
Spring	1497	38.3%	1			87	2.2%	1		
Summer	2829	52.8%	1.71	1.58–1.87	**p<0.001**	122	2.3%	1.01	0.76–1.33	p = 0.93
Autumn	1593	40.1%	1.06	0.97–1.17	**p = 0.19**	54	1.4%	0.60	0.43–0.85	**p = 0.004**
Winter	911	39.6%	1.00	0.90–1.11	**p = 0.98**	47	2.0%	0.92	0.64–1.31	p = 0.64

Chi-square analysis and logistic regression were used to compare BLL rate in subgroups. Odds ratios (OR), 95% confidence intervals (CI) and p-value were adjusted for potential confounding factors including age, sex and season. The first subgroup (boy group, 0–3 year group and Spring group) for each confounding factor was taken as control, and significant differences (p<0.001) were observed in some groups especially for the category >50 µg/L.

## Discussion

This study collected BLL data for all the out-patients from Wuhan Children Hospital in 2012 full year, and conducted an analysis of the data. The key findings in the study include: (a) BLLs found in this study were much lower than those reported in previous studies. (b) BLLs were higher in the boy group than the girl group. (c) BLLs were higher for the summer group, i.e., the group of the children who came to the hospital to measure BLLs in summer. (d) BLLs in different age groups were statistically different. Concretely, the BLLs for the toddlers were the lowest, went up in the preschool and school groups and gradually decreased in the teenagers. (e) For the preschool and primary school children (4–11 years old), 3,440 of the 6,567 children had BLLs higher than 50 µg/L, which means that lead poisoning prevention is still a serious problem, especially for the preschool and school children.

From the analysis, we found that only 2% of the children had BLLs higher than 100 µg/L, and the geometric mean of BLLs was 44.75 µg/L, which was much lower than those reported in previous studies. In comparison, Wang et al. [13] summarized 32 eligible articles in Chinese and reported that the mean of BLLs was 92.9 µg/L, and the prevalence of children with elevated BLLs was 33.8%. Compared with the BLLs in other cities of China, the BLLs in Wuhan were relatively low. In terms of the city of Wuhan itself, Qi et al. [16] showed that the mean of BLLs was 74.8 µg/L, and the prevalence of the children with elevated BLLs was 16.7% in 2002. Zhang et al. [15] reported that the mean of BLLs in Wuhan was 0.35 µmol/L (72.5 µg/L) in 2005, and the prevalence of mild poisoning was 17.4%. This showed that although the city of Wuhan had rapidly developed in industry, construction and urbanization in recent years, the BLL of the children in the city did not increase.

Regarding the relatively low BLLs, there are some possible explanations. First, the leaded petrol has been completely banned in China since 2000, and the amount of the lead in the air may decrease, which should be an important reason for the lower BLLs. Second, the problem of lead exposure has been recognized nationwide gradually. The community has been aware widely that lead may expose to children in multiple ways, including popcorn, puffed food, preserved eggs, canned food, sucking, nail-biting, eating paint, ink, crayons as well as home decoration. Currently, parents pay more attention to children's healthy eating and living habits, and tend to choose lead-free pencils and toys for kids. Furthermore, enterprises are also becoming aware of the hazards of leads, and more and more lead-free products are sold on the market. In addition, the Ministry of Health in China issued two articles titled “the Children Lead Acidosis and Lead Poisoning Prevention Guide" and "Children's Blood Lead and Lead Poisoning Classification Principles” in 2006. Since then, a wide range of health education on prevention of high blood lead and lead poisoning in children has been carried out. Public health staffs have used various methods, including face to face publicity and guidance, lectures, distributing promotional materials, etc., to spread knowledge about toxic effects of lead on children's development, and they have made great efforts to change parents' recognition, attitudes and behavior about lead poisoning. All these steps may be helpful to prevent and reduce the hazards of lead in children. Third, previous studies demonstrated that there is negative association between blood lead levels and nutrition, and if children suffer from malnutrition, especially when the body lacks calcium, iron, zinc and other elements, the absorption rate of lead increases. For example, the level of calcium and iron were supposed to affect lead absorption and further prevent lead poisoning [17]. So balanced diets and improvement of the nutritional status may also contribute to lower BLLs in children. Finally, since people did not fully realize the harm of lead poisoning in the past, only those children having obvious symptoms were taken to hospital to take the blood lead measurement. Nowadays, as clinical doctors and parents pay more attention to the problem, the BLL measurement is conducted more frequently.

From the analysis, we also found that boy group had higher BLLs than the girl group. The reason may be that boys normally have more outdoors activities, so they have more chances to lead exposure in the air, food, water or dust. In the mean time, we found that the pre-school and primary school children had higher BLLs than the children in other age stages. The possible explanation for this phenomenon is that the average height of pre-school and primary school might be the same as lead-contaminated zone in the air [13], [18], [19], and they may inhale more lead.

Further finding in this study includes the association between elevated BLLs and seasons: both the number of outpatients and the blood lead levels in summer were much higher than in other seasons. The possible reason for this may be due to the living environment and diet habit in summer. High temperature, humidity, air circulation and other weather-related factors in summer may increase the lead-contaminated dust in the air. On the other hand, the children usually have more out-door activities in summer, and excessive sweating can result in losing of a large number of trace elements and nutritional imbalance, which may induce the body to absorb more lead. In addition, some parents tend to use cool cornstarch and skin care products in summer, in which the main ingredient is talc powder, and the main component of talcum powder is lead. Lin et al reported a case of lead poisoning for two children caused by a kind of powder [20]. All these reasons might explain the elevated BLLs in summer.

In addition, we found that the blood lead levels in the toddle group were relatively low, which demonstrates that the elevated blood lead might not be related with mother-to-fetus or breastfeeding. It is known that the family planning policy has been carried out in China for a long time, and normally there is only one child in one family. Currently, experts strongly suggest that young couples ready to have children take blood lead measurement before pregnancy. This may be an important reason for lower blood lead in the toddler group.

As mentioned above, environment pollution and unhealthy living habits might be responsible for the level of blood lead. In addition, food safety may be another important factor for BLLs. Soil pollution by heavy metals including lead has been a main concern of the international communities recently, normally heavy metals from the soil can be taken by plants, and after biomagnifications in vivo, the concentration of the heavy metals in plants would increase, and finally lead may enter the body through food. So prevention of lead poisoning is a comprehensive process, which requires efforts and collaboration from many aspects in the community.

The limitation of this study lies in the following aspects. First, the data came from the out-patients in the children hospital, and only those children who were suspected of lead exposure by doctors were taken to measure their BLLs. Thus there existed a selection bias in this study, and the bias was towards higher BLLs. The real OR of the population should be lower. In this sense, the result can be seen as the maximal estimation of the children's BLLs in the period, and it only reflects the status of the BLLs to some extend. Second, understanding the affects of the related factors to lead poisoning is a crucial step in protecting children from the lead damage. In this paper, the association between BLLs and the risk factors including sex, age and season were explored. However, other risk factors for the elevated BLLs were not covered. In fact, the blood lead level may be due to varying factors, including environmental factors, unidentified genetic factors, socio-cultural variables, sex, race, feeding patterns, health habits, eating habits, family economic status and so on. Therefore, future work would include investigation of the role of these factors in lead poisoning.

In sum, the analysis suggests that BLLs of children in the city of Wuhan decreased recently. However, in comparison with the BLLs in other countries, the level in the city is still not satisfied. In US, according to National Health and Nutrition Examination Survey, the percentage of the children aged 1–5 years with BLLs ≥5* µ*g/dL in 1999–2002, 2003–2006, and 2007–2010 were 8.6%, 4.1% and 2.6% respectively, and the geometric mean of BLLs for these years were 19 µg/L, 16 µg/L and 13 µg/L respectively (7). In Canada, according to the Canadian Health Measures Survey (CHMS), geometric mean concentration in population was 13.4 µg/L, and less than 1% of the Canadians had high BLLs (>100 µg/L) from 2007 to 2009 [21]. In Asia, the geometric mean of BLLs about 10.7 µg/L was reported in Japan [22]. In comparison, 54% of the children in Bangladesh had high BLLs (>100 µg/L), and the mean of BLLs among the children was 134.5±82.1 µg/L in urban *vs*. 72.9±62.5 µg/dL in rural areas [23]. So in comparison with developed countries, there is still a long way for China cities and other developing countries to go on controlling lead pollution.

## Conclusions

From the results, we conclude that children's blood lead levels in Wuhan had obviously decreased in recent years; however, there were still 44% of the children having the elevated BLLs higher than 50 µg/L. So the situation is not optimistic. It is known that lead poisoning in children's development is a slow process, and there are no typical clinical manifestations in the early stage. Thus early detection to screen high lead hyperlipidemia in children and timely intervention to reduce the toxicity of lead on children's development are important and necessary. This study focused on the current situation of lead poisoning in Wuhan, which can provide support for local prevention and policy making. Lead exposure characteristics, lead poisoning molecular mechanisms, especially the mechanisms for its affect on children's brain development are all worth being understood in future, and the complete elimination of the impact of lead in our life is still a long term process.
